# Intravascular papillary endothelial hyperplasia (Masson’s tumor) of the finger: a case report and review of the literature

**DOI:** 10.1080/23320885.2025.2513066

**Published:** 2025-06-05

**Authors:** Malak Alsaif, Khalid Alosaimi, Turki Alhassan, Anas Alyamani, Mohammed Alfawzan

**Affiliations:** a Division of Plastic Surgery, Department of Surgery, King Abdulaziz Medical City, Ministry of National Guard - Health Affairs. King Abdullah International Medical Research Center, Riyadh, Saudi Arabia; b College of Medicine, King Saud University, Riyadh, Saudi Arabia

**Keywords:** Masson’s tumor, intravascular papillary endothelial hyperplasia (IPEH;), digital mass, hand tumors, hand mass, Saudi Arabia

## Abstract

Masson’s tumor, or intravascular papillary endothelial hyperplasia (IPEH), is a rare benign vascular lesion that can mimic other soft-tissue tumors. We present a case of a 33-year-old woman with a painful digital mass. Excision confirmed IPEH. Literature review identified eight similar cases, highlighting its rarity and diagnostic challenge.

## Introduction

Masson’s tumor, classically described as intravascular papillary endothelial hyperplasia (IPEH) and formerly known as ‘vegetant intravascular hemangioendothelioma,’ was first described by Masson in 1923 [1]. It is a benign, non-neoplastic overgrowth of vascular endothelium occurring as a reactive process following vascular injury. Masson’s tumor exhibits a rare incidence of approximately 2–4% of the benign and malignant vascular tumors of the skin and subcutaneous tissues, with a predilection for the head, neck, and upper extremities [2,3]. Globally, IPEH has been reported in various unusual anatomical locations, including the spine, foot, and mandible, highlighting its diverse presentations [3–5]. However, cases involving the hand or finger remain exceptionally rare, further contributing to the novelty of this case report. Clinically, IPEH can be challenging to identify due to its resemblance to other vascular and soft-tissue tumors. Imaging modalities, such as ultrasound and magnetic resonance imaging (MRI), play a role in raising suspicion for the diagnosis, but definitive confirmation relies on histopathological analysis [6]. Treatment involves complete surgical excision to prevent recurrence. Although IPEH is infrequently reported worldwide, documented cases from Saudi Arabia remain particularly rare. In 2020, a case was reported involving a 17-year-old female with a Masson’s tumor in the left metacarpophalangeal joint of the ring finger [7]. Recently, another case was reported involving the right thumb of a 69-year-old female [8]. Here, we present a rare case reported to be the second digital Masson’s tumor in Saudi Arabia and the third case involving the hand nationwide.

## Patients/materials and methods

**Figure 1.**
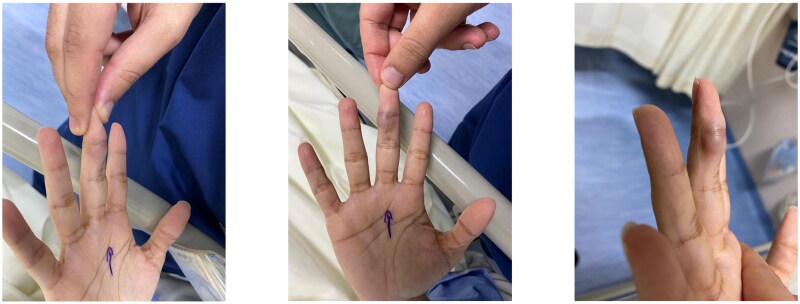
Pre-operative marking of the involved digit with Masson’s tumor.

A 33-year-old right-handed woman, with no significant past medical or surgical history, presented to our outpatient Plastic Surgery clinic with a chief complaint of a growing mass and swelling in her right middle finger. The patient reported moderate, intermittent pain associated with the mass over the past three months, with no history of major trauma or known inciting events. On physical examination, a round, cystic mass approximately 1.5 cm by 1.3 cm in size was observed on the volar-ulnar aspect of the digit, proximal to the distal interphalangeal joint (DIPJ), (Figure 1). The mass was non-mobile, non-transilluminating, and tender upon palpation. Despite the mass, the patient exhibited a full range of motion in the affected finger with no functional deficits, and the sensation was intact.

**Figure 2.**
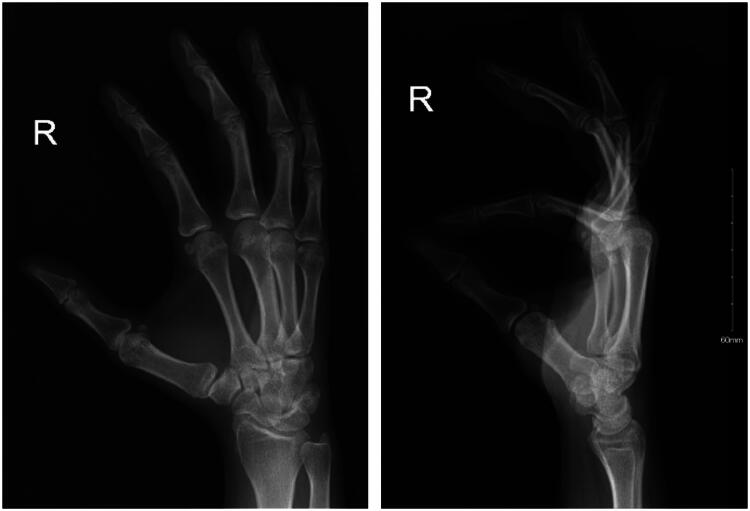
X-Ray images of the right hand in oblique, and lateral views.

**Figure 3.**
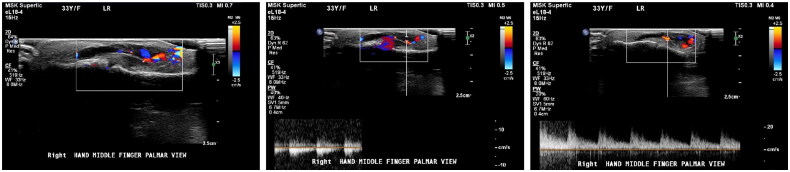
Ultrasound of the right Middle finger.

Initial X-ray imaging of the right hand revealed soft tissue swelling involving the volar aspect of the third middle and distal phalanx. No fractures or dislocations were noted (Figure 2). To further evaluate the mass, a right middle finger ultrasound was performed. The ultrasound demonstrated a subcutaneous superficial small heterogeneous cystic structure on the palmar aspect of the right middle finger, extending to the ulnar aspect and measuring approximately 2 × 0.4 cm. The lesion exhibited minimal peripheral vascularity, while the flexor tendon and volar plate remained intact. The initial impression suggested a phlegmon related to cellulitis, and clinical follow-up was recommended (Figure 3).

**Figure 4.**
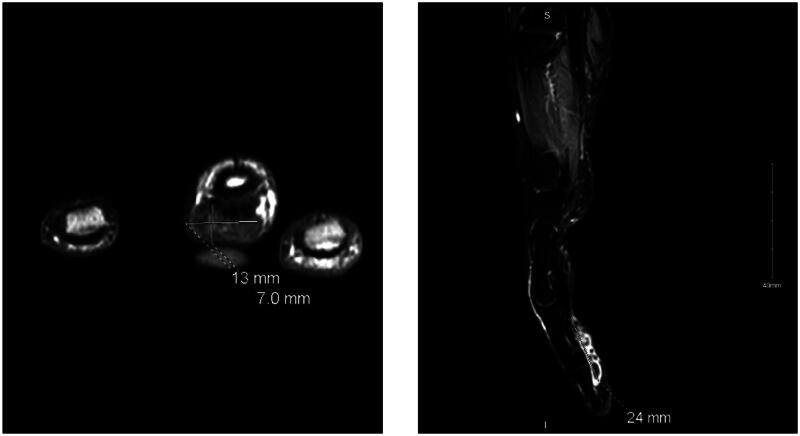
MRI of the right Middle finger.

Given the inconclusive findings, we proceeded with an MRI of the right hand, which provided a more detailed visualization. The MRI revealed a focal subcutaneous mass-like lesion located at the volar and ulnar aspect of the mid to distal third digit. The lesion, measuring 2.5 cm in its longest dimension by 1.2 × 0.7 cm in cross-section, was noted to have intermediate T1 and heterogeneous T2 signal intensity with heterogeneous enhancement. The mass was closely related to the adjacent flexor tendon and adherent to the tendon sheath, but no underlying bony abnormalities were observed (Figure 4). The differential diagnosis at this stage included an inflammatory phlegmon and a cancellous tumor of the tendon sheath, leading us to recommend an excisional biopsy for a definitive diagnosis.

**Figure 5.**
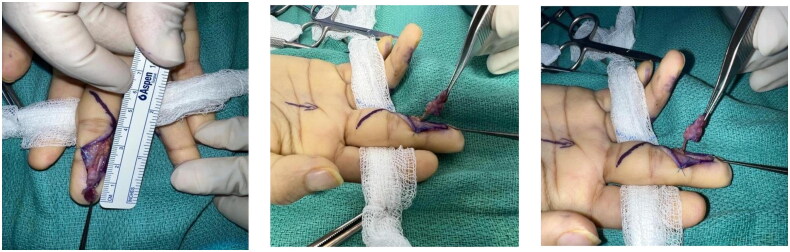
Intraoperative photograph showing the excised vascular mass with an identified feeding vessel, which was cauterized during surgery.

The patient was booked for an excisional biopsy two weeks later. During the surgery, the tumor was approached through a mid-lateral open incision. Careful dissection was performed, revealing the vascular nature of the mass, which was successfully excised along with the identified feeding vessel that was subsequently cauterized (Figure 5). The excised specimen was sent for histopathological analysis.

The surgical pathology report described the specimen as a 2.0 × 0.4 × 0.3 cm piece of gray, glistening soft tissue with a white cut surface and a rounded hemorrhagic tip measuring 0.5 cm at its maximum dimension. Histopathological analysis, including HHV-8 staining, confirmed features consistent with intravascular papillary endothelial hyperplasia (Masson’s tumor).

At her two-week follow-up appointment, the patient’s wound appeared clean with no signs of infection, and healing was progressing well. She exhibited a full range of motion in the affected finger with no functional deficits, and her sensation was intact. The patient was followed for 18 months postoperatively with no recurrence, after which she was lost to follow-up.

## Discussion

Masson’s tumor, also known as intravascular papillary endothelial hyperplasia (IPEH), is a benign, non-neoplastic proliferation of vascular endothelium that typically occurs as a reactive process following vascular injury. First described by Pierre Masson in 1923, IPEH represents approximately 2-4% of all benign and malignant vascular tumors of the skin and subcutaneous tissues [1,2]. While this condition has a predilection for the head, neck, and upper extremities, it remains a rare entity, with a limited understanding of its pathogenesis [3].

The development of IPEH is believed to be influenced by molecular and hormonal factors, which may explain the higher incidence of the tumor in females, with a reported ratio of 1.2:1 [9,10]. Unlike primary vascular tumors, IPEH arises within a thrombus, primarily in veins, where vascular injury, inflammation, and blood flow stasis activate chemotactic pathways that attract macrophages [11,12]. These macrophages release endothelial growth factors, stimulating tumor growth [11].

Histologically, Masson’s tumors are classified into three subtypes: (1) primary (de novo) arising from a normal blood vessel, (2) secondary (mixed) occurring in vessels with pre-existing pathological processes such as hemangiomas or pyogenic granulomas, and (3) an extravascular form that arises in hematomas [10]. These tumors are typically positive for CD31, CD34, and factor VIII-related antigen and negative for CD105, which helps differentiate them from angiosarcomas [13].

Clinically, Masson’s tumors present as small, well-defined, round to oval papules or nodules with a red or purple hue. They may be tender, as was the case with our patient, and can cause nerve compression symptoms if they arise near or involve the digital nerve vasculature [14]. These tumors are usually solitary but can be multiple within the involved area, growing slowly within the overlying skin, dermis, or subcutaneous tissue, with no reported incidence of malignant transformation [14]. The absence of malignant potential underscores the importance of accurate diagnosis to avoid unnecessary aggressive treatments.

Diagnosing Masson’s tumor can be challenging due to its resemblance to other vascular and soft tissue tumors, such as hemangiomas, pyogenic granulomas, vascular malformations, Kaposi’s sarcoma, epithelioid hemangioendothelioma and angiosarcomas [14]. Preoperative imaging, including ultrasound and MRI, can assist in the identification of these lesions, though their findings are not always definitive. In our case, the MRI findings of intermediate T1 and heterogeneous T2 signal intensity with enhancement were consistent with IPEH, but histopathological analysis ultimately confirmed the diagnosis, highlighting the indispensable role of tissue biopsy in the definitive diagnosis of Masson’s tumors.

The treatment of choice for Masson’s tumors is a complete surgical excision. This approach is crucial because incomplete excision may lead to recurrence, as documented in other cases [9,15]. Although the literature does not specify resection margins, a margin of 1 to 2 mm around the mass is generally recommended to minimize the risk of recurrence [13]. Our patient’s case was managed successfully with surgical resection, and the patient demonstrated good postoperative recovery with a full range of motion and no sensory or motor deficits, indicating a positive outcome. Notably, there was no recurrence at follow-up, which contrasts with some reports where recurrence occurred due to incomplete excision [9,15].

This case is unique not only because of its rarity as a digital Masson’s tumor, a presentation reported in only a handful of cases worldwide, but also because its clinical features were misleading and mimicked more common conditions. This overlap with diagnoses such as phlegmon or benign vascular lesions made the diagnosis challenging and highlights the importance of considering IPEH when evaluating digital masses. It represents the second reported case of a digital Masson’s tumor in Saudi Arabia and the third involving the hand nationwide. This adds to the limited but growing body of literature on IPEH in the region and provides valuable insights for clinicians encountering similar cases, particularly when clinical and imaging features suggest a vascular origin.

## Literature review

To contextualize our case, a focused literature review was conducted to identify reports of intravascular papillary endothelial hyperplasia (IPEH) involving the hand, and more specifically, the fingers. A search of PubMed, Scopus, and Google Scholar was performed using the terms ‘Masson’s tumor,’ ‘IPEH,’ and ‘digital mass.’ Articles published from inception to August 2024 were considered without language restrictions. After screening for relevance, eight cases of digital IPEH were identified and included. The key clinical features, diagnostic workup, and treatment outcomes from these cases are summarized in Table 1.

**Table 1.** The reported cases of intravascular papillary endothelial hyperplasia (masson’s tumor) of the digit in the literature.

First author/year	Country	Gender	Age (y)	Size (cm)	Location	Symptoms and Duration	Diagnostic Methods	Surgical approach	Complications
Lim, 2011 [16]	Singapore	Female	49	0.8 × 0.8	Volar ulnar border of the left index finger at the level of the proximal interphalangeal joint	Tender mass, decreased sensation on the ulnar half of the finger pulp, 3 years duration	US, MRI, Histopathology	Excisional biopsy under general anesthesia	None reported
Albazali, 2023 [17]	Kuwait	Female	67	0.2	Distal palmar aspect of the left thumb	Tender, firm bluish nodule, intact sensation, 2 months duration	No preoperative imaging, Histopathology (positive for CD31)	Excisional biopsy	None reported
Sung, 2021 [12]	Korea	Female	53	0.7 × 0.3 × 1.2	Volar surface of the proximal phalanx of the left index finger	Nontender, asymptomatic, protruding mass, intact sensation, 3 months duration	US, Histopathology (positive for CD31, and CD34)	Excision under digital nerve block, no feeding vessel was observed	None reported
Ng, 2020 [15]	Singapore	Male	19	2 × 1	Volar aspect of the proximal phalanx of the right little finger	Nontender, enlarging bluish nodule, with numbness, 3 months duration	US, Histopathology (positive for CD34)	Excision biopsy, the feeding vessel was identified and diathermised	Recurrence of the tumor 2 months postoperatively
Manafi, 2022 [18]	Iran	Male	30	2 × 0.9	Volar aspect of the distal phalanx of the index finger of the left hand	Tender bluish mass, 8 months duration	Plain X-ray, MRI, Histopathology	Excisional biopsy	None reported
Mitchell, 2021 [19]	USA	Male	48	1.5	Dorso-radial aspect of the proximal phalanx of the right index finger	Nontender, painless, freely mobile swelling, 2 months duration	US, Histopathology	Excisional biopsy	None reported
Hutcheson, 2012 [20]	USA	Female	63	2.4	Ulner aspect of the left thumb	Painless mass, with decreased sensation, several years duration	No preoperative imaging, Histopathology	Excisional biopsy with concomitantneuroplasty of the digital nerve	None reported
Alkabbaa, 2023 [8]	Saudi Arabia	Female	69	1 × 1	Dorsal side of the right thumb over the metacarpophalangeal joint level	Round, nontender mass, with intact sensation, 1 year duration	Duplex US, Histopathology	Excisional biopsy, two feeding vessels located and ligated	None reported

These reports collectively underscore the rarity of IPEH in the fingers. Most lesions presented as slowly enlarging, tender nodules, often resembling other benign vascular lesions. MRI frequently contributed to the preoperative assessment, while definitive diagnosis was established histologically, helping to distinguish IPEH from malignant entities such as angiosarcoma. Despite these contributions, the existing literature remains limited, with considerable variability in surgical margins, follow-up protocols, and long-term outcome reporting. There is a clear need for larger, multicenter studies to better define the clinical course of IPEH and to develop standardized diagnostic and management strategies. Furthermore, future research may benefit from incorporating molecular and genetic analyses to improve understanding of this uncommon vascular entity, particularly in rare anatomical locations such as the fingers.

## Conclusion

Masson’s tumor, though rare, is a crucial consideration in the differential diagnosis of hand and digital masses, given its potential to mimic more aggressive conditions. This case underscores the vital role of histopathological analysis in confirming the diagnosis and guiding treatment. For plastic surgeons, recognizing and accurately diagnosing Masson’s tumor is essential to ensure appropriate management and avoid unnecessary aggressive interventions.
